# Chelating Agent Functionalized Substrates for the Formation of Thick Films *via* Electrophoretic Deposition

**DOI:** 10.3389/fchem.2021.703528

**Published:** 2021-06-17

**Authors:** Sara C. Mills, Natalie E. Starr, Nicholas J. Bohannon, Jennifer S. Andrew

**Affiliations:** Department of Materials Science and Engineering, University of Florida, Gainesville, FL, United States

**Keywords:** nanomaterials, assembly, electrophorectic deposition, magnetic nanopartcles, chelating ligands

## Abstract

Incorporating nanoparticles into devices for a wide range of applications often requires the formation of thick films, which is particularly necessary for improving magnetic power storage, microwave properties, and sensor performance. One approach to assembling nanoparticles into films is the use of electrophoretic deposition (EPD). This work seeks to develop methods to increase film thickness and stability in EPD by increasing film-substrate interactions *via* functionalizing conductive substrates with various chelating agents. Here, we deposited iron oxide nanoparticles onto conductive substrates functionalized with three chelating agents with different functional moieties and differing chelating strengths. We show that increasing chelating strength can increase film-substrate interactions, resulting in thicker films when compared to traditional EPD. Results will also be presented on how the chelating strength relates to film formation as a function of deposition conditions. Yield for EPD is influenced by deposition conditions including applied electric field, particle concentration, and deposition time. This work shows that the functionalization of substrates with chelating agents that coordinate strongly with nanoparticles (phosphonic acid and dopamine) overcome parameters that traditionally hinder the deposition of thicker and more stable films, such as applied electric field and high particle concentration. We show that functionalizing substrates with chelating agents is a promising method to fabricate thick, stable films of nanoparticles deposited *via* EPD over a larger processing space by increasing film-substrate interactions.

## 1 Introduction

For applications that utilize films of nanoparticles, there is often a need to develop methods to fabricate films that are both thick and stable. For example, better film-substrate stability can improve upon flexible materials such as electronics and wearable devices, increase durability, and enhance corrosion protection (Ming et al., 2014; Sun et al., 2015; Ramírez-García et al., 2017). Along with film-substrate stability, increasing film thickness can improve a material’s performance in microwave applications, energy storage capabilities, and optical and sensing properties (Rao et al., 2016; Verma et al., 2018; Cao et al., 2018; Mahender et al., 2019). There are a number of nanoparticle deposition techniques that can be used to fabricate nanoparticle films, such as drop-casting, spin-coating, dip-coating, spray-coating, aerosol deposition, electrophoretic deposition, and Langmuir-Blodgett deposition, to name several (Niederberger, 2017). One of the aforementioned methods that has received increasing attention for depositing nanoparticle films with controllable thickness and relative ease is electrophoretic deposition (EPD) (Amrollahi et al., 2015).

EPD is a colloidal technique that moves charged particles towards an oppositely charged electrode under an applied external field, resulting in particle deposition and film formation (Ferrari and Moreno, 2010). Increasing the thickness and stability of films deposited *via* EPD has been traditionally accomplished through the addition of dispersing agents and/or tuning the deposition parameters. However, the use of dispersing agents can introduce additional considerations of chemical compatibility between the additive and solvent and non-ideal behavior in solution, while particular deposition parameters can introduce unfavorable particle-substrate interactions and non-ideal particle behavior in solution. Ultimately, these approaches can limit the maximum achievable yield (Yum et al., 2003; Besra and Liu, 2007; Khalili et al., 2016; Liu et al., 2016; Dhiflaoui et al., 2017).

**FIGURE 1:**
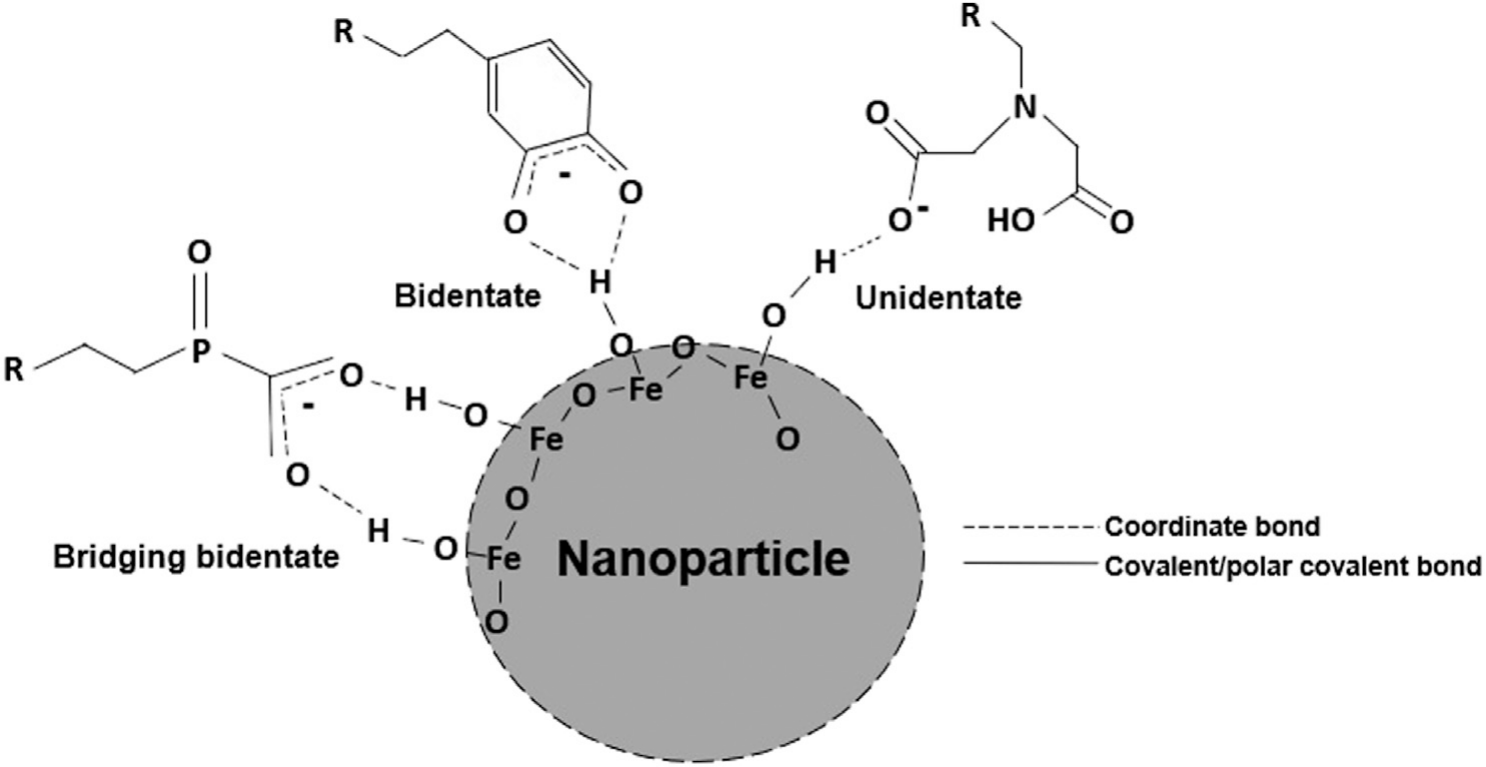
Schematic illustrating coordination bonding between chelating agents of various dents and the hydrogens of the surface −OH (hydroxyl) groups of a metal oxide nanoparticle.

**FIGURE 2:**
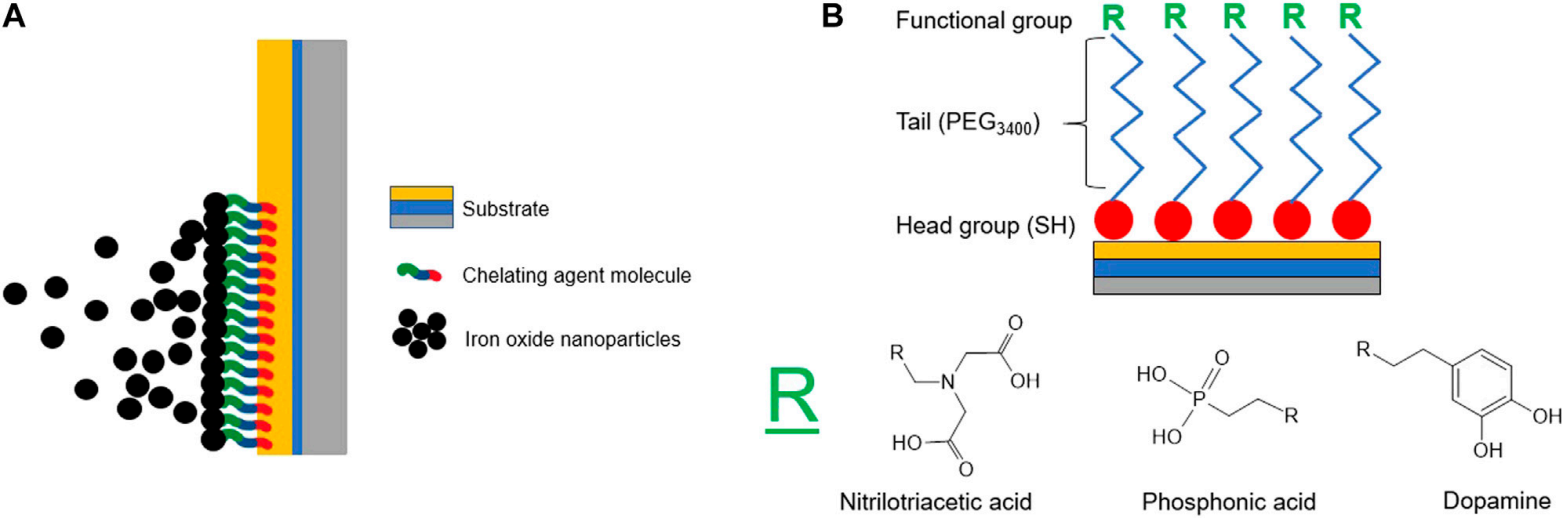
**(A)** Illustration of the substrate functionalized with chelating agent molecules for use during the electrophoretic deposition of iron oxide nanoparticles. **(B)** Expected structure of the chelating agent molecules as they are functionalized on an Au/Ti/Si substrate, including a thiol head group, a PEG3400 spacer, and functional group (R).

To overcome the limitations of existing EPD methods, we have developed an approach that functionalizes EPD substrates with chelating agents promoting stronger film-substrate interactions, which can be leveraged to ultimately fabricate thick films. Chelating agents have the ability to electrostatically interact with the hydrogens of the -OH groups on the surface of metal oxide nanoparticles (Bonvin et al., 2017). Chelating agents (Figure 1) are classified based on the number of dents, or coordination sites, their functional groups possess. As the number dents increases, so does the possibility of more hydrogen interactions, increasing the strength of the overall interaction (Walter et al., 2015). If the functional group of the chelating agent consists of an electron-donating species or can form a resonant structure, the interaction can be stabilized further with σ*-* and π*-*bond donation (Turcheniuk et al., 2013). In this work, the effect that substrates functionalized with chelating agents with different functionalities have on film thickness and stability when deposited with two different EPD parameters sets will be studied *via* the deposition of iron oxide nanoparticles by EPD, as shown in Figure 2A.

Here, to test the effect of different chelating agents we selected three functional groups that have been extensively utilized in the surface functionalization of iron oxide nanoparticles due to their relatively strong coordination to the nanoparticle surface (Boyer et al., 2010; Walter et al., 2015). Specifically the functional groups include carboxylic acid, phosphonic acid, and catechol *via* nitrilotriacetic acid (NTA), phosphonic acid (PA), and dopamine (DPA) based chelating agents, respectively. Additionally, these functional groups have differing coordination strengths based on the electronegativity and electron distribution of the group and a different amount of coordination sites (Boyer et al., 2010; Walter et al., 2015; Bonvin et al., 2017). Experimentally, coordination strengths of the chosen functional groups to magnetic nanoparticles have been measured *via* various methods, including the rate of grafting to the nanoparticle surface, spectroscopic studies, and Langmuir isotherms (Chen et al., 2002; Xu et al., 2004; Daou et al., 2007; Daou et al., 2008). Functional groups that have higher grafting rates to magnetic nanoparticles, such as those that contain phosphonate groups (PA), have stronger bonds to the nanoparticle surface when compared to those with carboxylic acid groups (such as NTA) (Daou et al., 2007, 2008). With respect to the third chelating agent, DPA, tight bonding to the surface of iron oxide nanoparticles has been demonstrated *via* structural changes upon DPA binding as well as the demonstration of favorable adsorption of DPA to the surface of magnetic nanoparticles when compared to desorption (Chen et al., 2002; Xu et al., 2004). These three chelating agent functional groups were ultimately functionalized on the surface of gold substrates by utilized the affinity of thiol groups (-SH) for gold, shown in Figure 2B (Vericat et al., 2010). This thiol head group will be bonded to a functional tail group, consisting of the following: a polymer spacer group of poly (ethylene glycol) (PEG3400) which will then be bonded to three different chelating agents: nitrilotriacetic acid (NTA), phosphonic acid (PA), and dopamine (DPA).

The hypothesis is that chelating agents can be used to form thicker films *via* EPD by improving substrate-film interactions. The chelating agents on the surface of the substrate will promote a more stable first layer of particles, improving the interactions between the substrate and film. The next incoming layer of particles will interact with the stable first layer *via* van der Waals interactions. However, because of the improved interaction between the first layer of deposited particles and the substrate, thicker films can be achieved when compared to traditional, non-functionalized substrates. According to the Hamaker equation (Eq. 1), which shows the effects of several factors and their effect on deposition yield, substrate-film interactions, most commonly discussed as adhesion, is represented by the sticking factor (*f*).

$$w=\int_{t_{1}}^{t_{2}}f\mu EAC\text{d}t$$ (1)

In this equation, the yield of the deposit (*w*) is related to the following parameters: the strength of the electric field (*E*), electrophoretic mobility of the particles (*μ*), surface area of the electrode (*A*) and the concentration of particles in solution (*C*) over a particular amount of time (*t*) (Besra and Liu, 2007). Substrates functionalized with chelating agents will promote thicker films by increasing the sticking factor, *f*, by increasing the interactions between the first layer of deposited particles and the substrate. After functionalizing substrates with each chelating agent, two sets of EPD parameters were tested to study the effect that functionalization has on film thickness, yield, and stability. The two particular EPD parameters chosen were the concentration of particles in solution and applied field, two parameters that should theoretically increase yield (*w*). When increased in practice, however, they do not lead to reliable film formation due to suspension turbidity and agglomeration (Besra and Liu, 2007; Amrollahi et al., 2015). Here, we compare parameters that reliably form films (moderate field and low particle concentration) against conditions that hinder dependable film formation (high field and high particle concentration).

This study investigates the ability of chelating agent functionalized substrates to enhance film thickness and stability using electrophoretic deposition. We explore (1) the effect that functional groups with a higher affinity and bond strength with the iron oxide nanoparticle surface have on increasing film thickness and (2) the ability of substrates functionalized with chelating agents to improve film stability and thickness, particularly with EPD parameters that do not normally produce reliable films. For example, deposition parameters such as high particle concentration and high applied electric field should theoretically increase yield (see Eq. 1), but do not do so in practice. However, films deposited on substrates functionalized with chelating agents can overcome these parameters that do not typically produce stable, thick films. Overall, this surface functionalization method is a versatile approach that can be to applied to other metal oxide nanoparticle systems, providing an innovative way to enhance the deposition of films *via* EPD. This approach has the ability to be employed in the fabrication of high-quality nanoparticle films. Applications that require the fabrication of thick and stable nanoparticle films but suffer from fabrication limitations could highly benefit from this substrate functionalization method, as it can be easily tailored to various substrates and particle systems.

## 2 Materials and Methods

### 2.1 Materials

Thiol-poly (ethylene glycol)-N-Hydroxysuccinimide and thiol-poly (ethylene glycol)-nitrilotriacetic acid (MW 3400 Da) were purchased from Biochempeg Scientific, Inc. (Watertown, MA). 2-aminoethylphosphonic acid and dopamaine hydrochloride were purchased from Sigma Aldrich, Inc. (St. Louis, MO). All organic solvents [N-methylmorpholine (99%), 200-proof ethanol (99.5%, ACS), acetone (ACS), isopropanol (ACS), tetraethylammonium hydroxide (10% in water), and N,N-dimethylformamide (99.8%)], iron (III) chloride hexahydrate, iron (II) chloride hexahydrate, regenerated cellulose dialysis tubing [6,000–8,000 Da, and 3,500 Da Molecular Weight Cut-Off (MWCO)], HEPES [4-(2-hydroxyethyl)-1-piperazineethanesulfonic acid] buffer (1 M, pH 8), and hydrochloric acid were purchased from Fisher Scientific.

### 2.2 Methods

#### 2.2.1 Nanoparticle Synthesis and Stock Solution Preparation

Iron oxide nanoparticles were synthesized *via* aqueous co-precipitation by combining iron (II) chloride tetrahydrate and iron (III) chloride hexahydrate in a molar ratio of 1:2. This synthesis was adapted from a previously reported method Massart. (1981). Briefly, the iron (II) and iron (III) chloride salts were reacted in an alkaline solution (between pH 8–9) for 1 h at 85°C. After 1 h, the particles were collected with a permanent magnetic, washed, and dialyzed to remove excess salts using 6,000–8,000 Da MWCO dialysis tubing. After dialysis, the particles were collected with a magnet, washed again, and peptized in deionized water along with tetrethylammonium hydroxide (TEAH) to create a colloidally stable suspension of particles, which served as a stock solution. The final concentration of the stock suspension was determined by freeze drying three 1 ml aliquots and recording the mass of the dried particles. To isolate the effect of surface functionalization on yield a new stock suspension was prepared for each surface treatment as well as for each set of experimental conditions. The average concentration of each of the final stock suspensions was adjusted to 46.57 ± 3.4 mg/ml.

#### 2.2.2 Characterization of Nanoparticles

##### 2.2.2.1 X-Ray Diffraction

The crystalline phase of the synthesized iron oxide nanoparticles was analyzed *via* X-ray diffraction (XRD). The powder samples were scanned using a Panalytical X’pert powder diffractometer, using a Cu anode (K*α* radiation). This instrument employed a scintillation detector (45 kV, 45 mA) with a step size of 0.008°. The phase was confirmed by comparing to a magnetite (Fe_3_O_4_) reference diffraction pattern [98-004-4525 from the International Center for Diffraction Data (ICDD)]. Scherrer’s formula was used to calculate crystallite size (*τ*) *via* the equation $\tau =\frac{k\lambda }{\beta \cos\theta }$, where k is a shape factor (0.9 for spherical particles), *λ* is the X-ray wavelength (1.54 Å), β is the full width half maximum value (corrected for instrument broadening, in radians), and θ is the Bragg angle, also in radians.

##### 2.2.2.2 Transmission Electron Microscopy

The physical size of the iron oxide nanoparticles was measured using a 100 kV Hitachi H7000 transmission electron microscopy (TEM). A dilute suspension of the nanoparticles in water was drop-casted onto a formvar-coated copper mesh grid for imaging. ImageJ software was used to measure the diameter of the particles (*n* = 250 particles).

#### 2.2.3 Chelating Agent Synthesis

**FIGURE 3:**
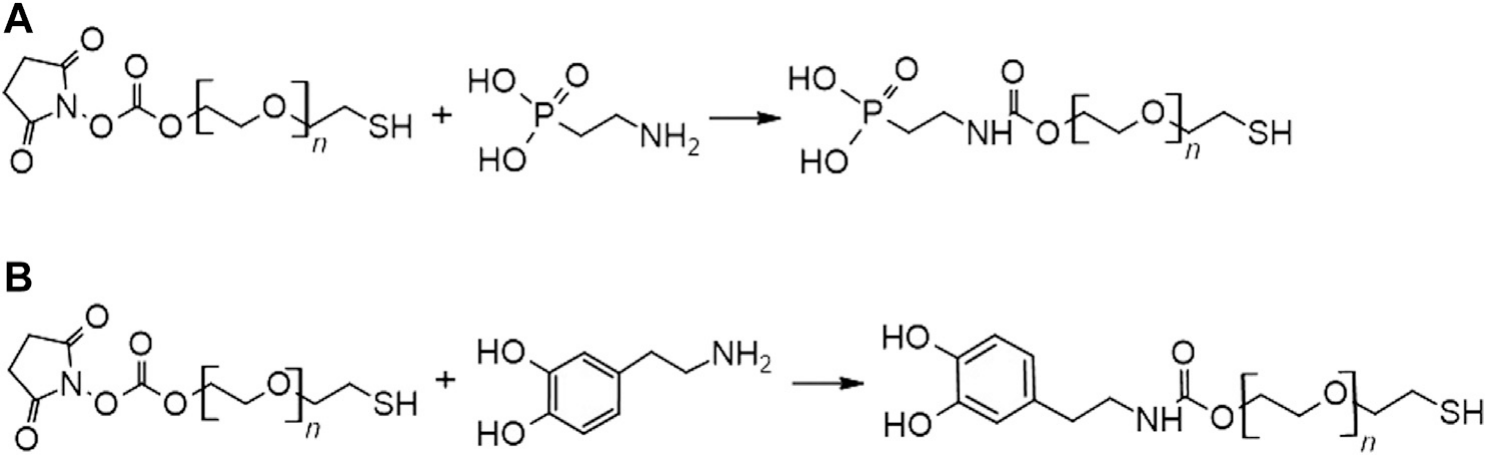
Reaction scheme for synthesizing **(A)** SH-PEG-PA and **(B)** SH-PEG-DPA (*n* = 3,400).

The synthesis of SH-PEG-PA and SH-PEG-DPA was accomplished with synthetic routes that utilized the reactivity of primary amines with N-hydroxysuccinimide (NHS)-ester groups, as shown in Figure 3.

##### 2.2.3.1 SH-PEG-PA

To synthesize SH-PEG-PA, 2-aminoethylphosphonic acid was added to HEPES buffer (1 M, pH 8) in water and was stirred to ensure complete dissolution. In a separate vessel, SH-PEG-NHS was dissolved in N,N-dimethylformamide (DMF) by mixing and applying low heat to increase solubility. The dissolved SH-PEG-NHS in DMF was added to the 2-aminoethyl phosphonic acid solution in a 10:1 ratio of 2-aminoethyl phosphonic acid to SH-PEG-NHS (1.2:0.12 mmol). This reaction was carried out for 5 h at room temperature. The product was then transferred into 3,500 MWCO dialysis tubing and was dialyzed for 72 h, changing the water after 4 h initially and then every 18 h. After dialysis the product was freeze dried.

##### 2.2.3.2 SH-PEG-DPA

The synthesis of SH-PEG-DPA was adapted from a previously reported method Xu et al. (2017). Dopamine hydrochloride (0.07 mmol) and SH-PEG-NHS (0.08 mmol) were dissolved separately in DMF. The SH-PEG-NHS was stirred and heated slightly to increase solubility. N-methylmorpholine (0.1 mmol) was added to the dopamine solution and was stirred under nitrogen for 25 min. The dopamine solution was then added to the SH-PEG-NHS solution and was left to react overnight at room temperature under low stirring. The product was then diluted with deionized water to bring the theoretical concentration to between 15–20 mg/ml. The diluted product was then transferred to 3,500 MWCO dialysis tubing and placed in a pH = 3 dialysis bath (0.001 M HCl in deionized water) for 48 h, changing the bath after 4 h, then at 24 h intervals. After 48 h, the product was transferred to a bath of deionized water for 2 h to remove any remaining acid. The product was then freeze dried.

#### 2.2.4 Characterization of SH-PEG-PA and SH-PEG-DPA

The structure of the synthesized chelating agents was analyzed with Fourier Transform Infrared (FTIR) spectroscopy with an attenuated total reflectance (ATR) accessory. The freeze dried chelating agent was loaded onto the ATR window of a Thermofisher Nicolet 6,700 spectrometer. The spectra were collected with 4 cm^−1^ resolution and 64 scans per sample.

#### 2.2.5 Substrate Fabrication

Conductive substrates were fabricated to provide the deposition electrode. The substrates were fabricated from a 100 p-type silicon wafer (100 mm diameter). A 10 nm titanium adhesion layer was sputtered onto the silicon substrate, followed by a 100 nm gold seed layer using a KJL CMS-18 Multi-Source sputtering tool. Substrates were then cut using a dicing saw to a size of 6 mm × 20 mm.

#### 2.2.6 Substrate Functionalization

Prior to functionalization, substrates were sequentially cleaned with acetone, isopropanol, and deionized water. This sequence was performed three times. The substrates were then dried with nitrogen after the third deionized water wash. The solutions for functionalization consisted of a 1 mM solution of the desired chelating agent in ethanol. This solution was stirred and heated slightly to fully dissolve the chelating agent. The cleaned substrate was then submerged in the chelating agent solution under constant stirring. Evaporation was prevented by covering the reaction vessel with parafilm. Functionalization was carried out for 24 h, during which the thiol groups of the dissolved chelating agent reacted with the gold surface to form self assembled monolayers of the chelating agents as illustrated in Figure 2B. After 24 h, the substrate was rinsed with deionized water three times, and dried gently with nitrogen. The substrate was then stored in a vacuum oven at 40°C prior to electrophoretic deposition.

#### 2.2.7 Characterization of Functionalized Substrates

The surfaces of the functionalized substrates were analyzed to detect that the thiol of the chelating agents had reacted with the gold surface with X-ray photoelectron spectroscopy (XPS) using a ULVAC-PHI VersaProbe II XPS (Physical Electronics, Inc.). A monochromated X-ray beam from an aluminum target (25 W) was focused 45° relative to the sample surface. Survey scans were performed at 93.9 eV and high resolution spectral scans were obtained at 23.5 eV. A step size of 0.8 eV was used for the survey spectrum with each spectrum being collected five times. High resolution spectral scans used a step size of 0.1 eV and each peak was scanned 10 times.

#### 2.2.8 Electrophoretic Deposition

For electrophoretic deposition, an iron oxide nanoparticle suspension was prepared by adding the iron oxide nanoparticle stock suspension at either one or 5 vol% to a 0.01 M hydrochloric acid (HCl) in isopropanol (IPA). This EPD suspension yielded an effective pH (pH_*e*_) of 2.3, providing the particles with a highly positive surface charge (approximately +30 mV). In the EPD bath a graphite block (40.1 × 26.1 × 3.3 mm) was used as the positively charged electrode, and was placed parallel and 1.5 cm away from the substrate. A power supply (Bio-Rad PowerPac HV) was used to generate an electric field between the two electrodes, with the positive electrode connected to the graphite block (counter electrode) and the negative electrode connected to the substrate, resulting in the particles moving towards and depositing on the substrate. The specific field and time deposition conditions are detailed in Table 1. After EPD, the deposited films were allowed to dry in air overnight.

**TABLE 1:** Experimental conditions for EPD.

	Moderate field and low particle concentration	High particle concentration and field
HCl in IPA [M]	0.01	0.01
Applied field (V/cm)	30	80
Stock suspension concentration (vol%)	1	5
Particle concentration (vol%)	0.009	0.04
Time (min)	45	30

#### 2.2.9 Characterization of Film Thickness

Deposited films of iron oxide nanoparticles on Au/Ti/Si substrates were prepared for cross sectional scanning electron microscopy (SEM), by first mounting each substrate onto a glass slide (25.4 × 25.4 mm) with double sided tape. A poly (vinyl) alcohol layer was then spin coated, with a G3P Spincoater (Speciality Coating Systems, Inc.), onto the films so the film would stay intact during cleaving for cross-sectional SEM. A 1 wt% PVA solution was prepared in water and dispensed onto the film, and was spin coated at 1,500 rpm. After spin-coating was complete, the substrates were removed from the glass slide and were cleaved and mounted on 45° stubs.

Cross-sectional SEM was performed on the deposited films with a FEI Nova NanoSEM 430 at an accelerating voltage of 5 keV. Each substrate was tilted to 45–50°. A total of 10 images were taken of each deposited film at different regions across the entire film. The acquired images were analyzed with ImageJ software, with 10 measurements of the cross-sectional thickness per image, resulting in 100 measurements per film. For each set of experimental conditions, three samples were made, resulting in 300 measurements for each experimental group.

#### 2.2.10 Adhesion and Mass Lost Tests

To investigate the effect that surface functionalization has on film stability and adhesion for the high particle concentration and high applied field condition, the Tape Test (from ASTM-D3359-17: Standard Test for Rating Adhesion) was performed on non-functionalized substrates and those functionalized with SH-PEG-DPA ASTM (2017). After deposition, films were placed in a 40°C vacuum oven for 72 h to ensure the film was dry. Briefly, the Tape Test was performed using Test Method A, wherein an X-cut was made with a sharp razor blade through the deposited film, exposing the gold surface of the substrate. To make the X-cut, two lines of approximately 9 mm in length were cut with the razor blade, using the straight-edge of a ruler to ensure straight lines. Scotch-brand pressure-sensitive tape was then applied over the entirety of the cut. Pressure was applied to the surface of the tape to ensure contact between the tape and film and was removed after the tape had been applied for 1 min.

After the adhesion tests were complete, the masses of the remaining film and substrate were measured. This was in order to investigate the ability of the functionalized films to retain particles after the adhesion test when compared to the non-functionalized films. These mass loss results provide a more quantitative approach to the Tape Test, and compliment the observed instability of non-functionalized films deposited under the high particle concentration and high applied electric field condition.

## 3 Results and Discussion

### 3.1 Iron Oxide Nanoparticle

The iron oxide nanoparticles utilized for this study were characterized in our previous work (Mills et al., 2020). Briefly, XRD patterns of the as-synthesized particles were compared with a reference magnetite (Fe_3_O_4_) pattern (ICDD pattern 98-004-4525). Peaks attributed to inverse spinel crystal structure at 2*θ* = 30.1, 35.8, 53.4, 57.1, and 62.9° confirmed the presence of inverse spinel iron oxide, which was expected from this synthesis (Supplementary Figure S1A). The physical size of the particles was obtained by measuring 250 particles from TEM images. The average particle size was 10.6 ± 2.4 nm (Mills et al., 2020). Crystallite size was calculated *via* Scherrer’s formula, using three 2*θ* values for the calculations: 35.8, 57.1, and 62.9°. The average crystallite size was calculated as 13.5 ± 1.3 nm, in good agreement with the TEM results.

### 3.2 Chelating Agent Synthesis

**FIGURE 4:**
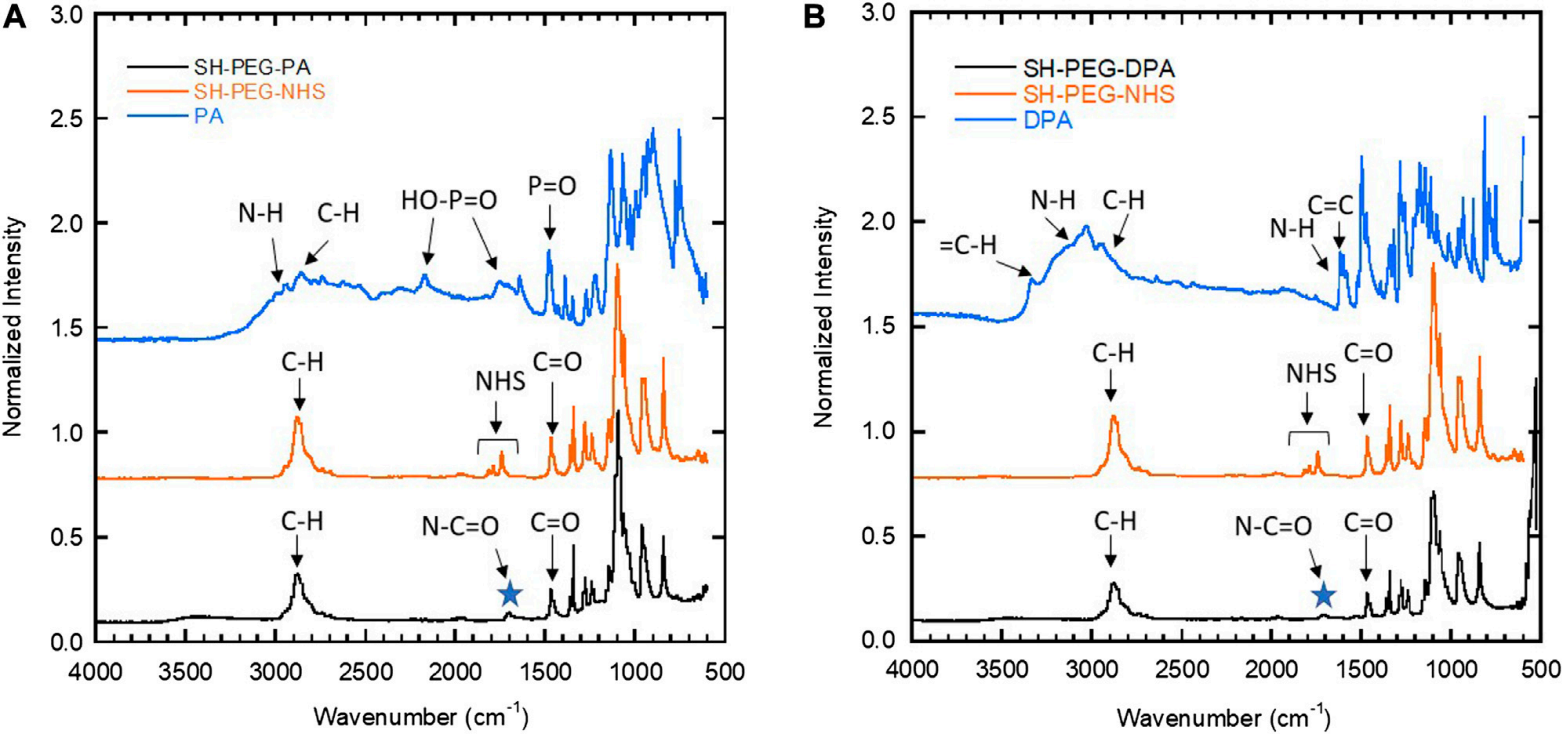
FTIR spectra of the two starting materials and the resulting chelating agent molecule for **(A)** SH-PEG-PA and **(B)** SH-PEG-DPA. Both **(A)** and **(B)** show the presence of an amide bond at 1700 cm^−1^ (noted by the star) and the disappearance of the peaks associated with the NHS group, seen around 1750–1850 cm^−1^ in the SH-PEG-NHS spectra but not in the final product in **(A)** or **(B)**.

FTIR measurements (Figure 4) confirmed the structure of the synthesized chelating agents (SH-PEG-PA and SH-PEG-DPA). FTIR is a technique that probes the characteristic stretching and bending of functional groups, and was used to confirm the presence of an amide bond in the final product. This amide bond (O=C-N) has a backbone stretch, which appears at approximately 1700 cm^−1^, and indicates that the primary amine of the two starting materials (2-aminoethylphosphonic acid and dopamine hydrochloride for the SH-PEG-PA and SH-PEG-DPA, respectively) have reacted with SH-PEG-NHS (Figure 3). The FTIR spectra for both SH-PEG-PA and SH-PEG-DPA (Figure 4) show the presence of an amide bond, as well as the disappearance of the peaks attributed to the NHS functional group (1750–1850 cm^−1^). The FTIR spectrum of the as-received SH-PEG-NTA can be seen in Supplementary Figure S2.

### 3.3 Substrate Functionalization

The substrates functionalized with SH-PEG-NTA were analyzed *via* XPS (Supplementary Figure S3), where the S2p spectrum was used to determine the presence of sulfur from the thiol group, along with evidence of whether the thiol reacted with the gold surface of the substrate. The peak centered around 163 eV can be attributed to sulfur. The two peaks within the envelope (162.5, and 164.4 eV) (Supplementary Figure S3B) arise from the presence of both chemisorbed and adsorbed thiol, respectively (Vericat et al., 2010; Spampinato et al., 2016). The C1s spectra (Supplementary Figure S3C) has also been analyzed, showing the presence of C-C/C-H bonds (285.5 eV) and C-O bonds (287.1 eV) attributed to the PEG of the polymer spacer chain of the chelating agent molecule. The presence of chemisorbed thiol as well as the characteristic bonds from PEG show that SH-PEG-NTA has successfully functionalized the gold substrate.

### 3.4 Effect of Surface Functionalization on EPD Films

**FIGURE 5:**
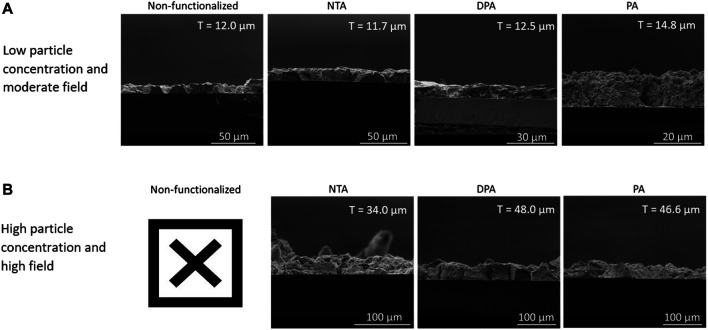
Panel of cross-sectional SEM micrographs showing the films for substrates that are non-functionalized and those functionalized with NTA, DPA, or PA for the **(A)** low particle concentration and moderate field and **(B)** high particle concentration and high field conditions. The “X” in panel **(B)** indicates that cross-sectional SEM images were not able to be obtained for the non-functionalized samples. The average thickness of each film is indicated by “T” in the top-right corner of each SEM image.

The effect that functionalizing the surface of substrates used for EPD with chelating agents has on film thickness and stability was tested using two sets of experimental conditions, seen in Table 1. The two sets of conditions were chosen to represent parameters that traditionally lead to reliable film formation (low particle concentration and moderate field) and those that should increase yield but do not in practice due to agglomeration and suspension turbidity (high particle concentration and high field). Figure 5 shows cross-sectional SEM micrographs of the films for non-functionalized and functionalized substrates from the low particle concentration, moderate field (Figure 5A) and high particle concentration, high field (Figure 5B) EPD conditions for each chelating agent. Supplementary Figure S4 shows additional, higher magnification SEM images comparing the microstructure of the nanoparticle films between non-functionalized and functionalized films for the first set of conditions. Both the non-functionalized and functionalized films show good morphology, indicating that the surface functionalization does not lead to a difference in microstructure. The first set of deposition conditions, low particle concentration and moderate field, was chosen because that stock suspension concentration (1 vol%) and applied electric field (30 V/cm) resulted in the reliable deposition of films in our previous work, which represents traditional EPD conditions (Mills et al., 2020). The other set of conditions, high particle concentration (5 vol%) and high field (80 V/cm) provided the opportunity to evaluate the ability of the chelating agents to improve film thickness and stability *via* EPD conditions that do not traditionally deposit reliable films. If these conditions did in fact reliably deposit films, Eq. 1 (Hamaker’s equation) dictates that the yield of the deposit should significantly increase, which is often directly correlated to an increase in film thickness.

**FIGURE 6:**
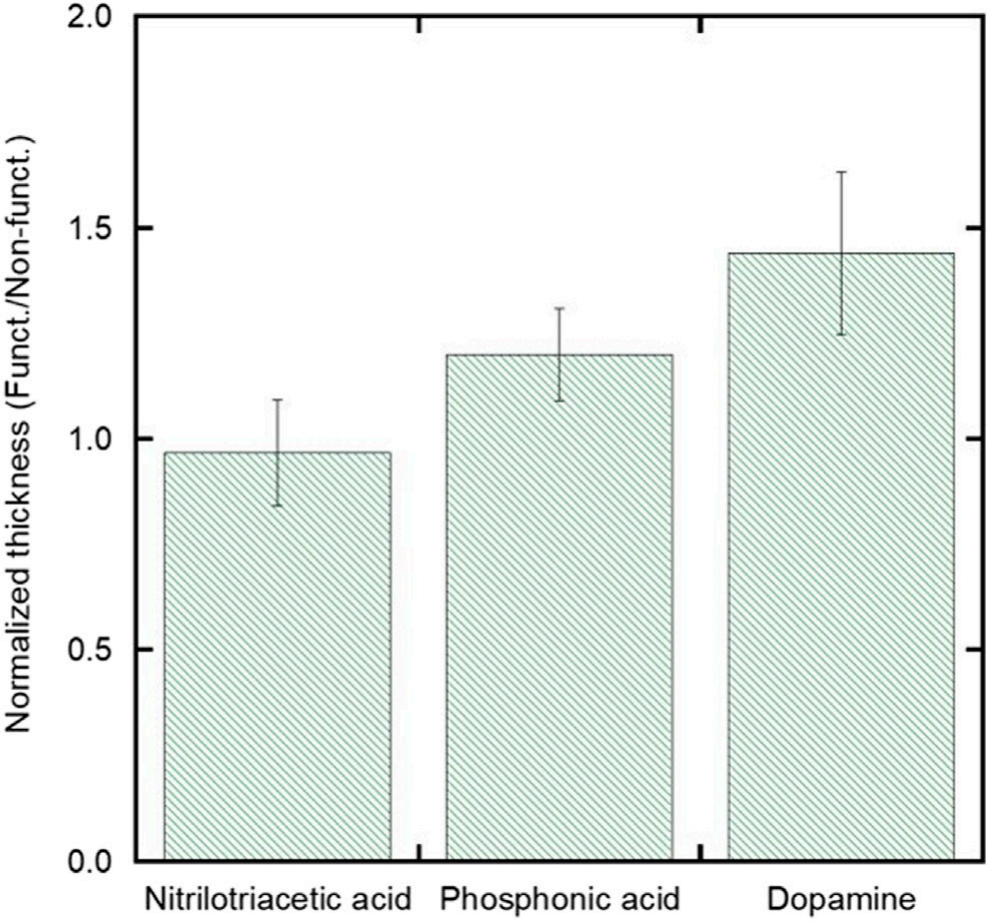
Plot showing the normalized thicknesses (defined as the average functionalized thickness divided by the average non-functionalized thickness) comparing the films that were both non-functionalized and functionalized with NTA, PA, or DPA for the low particle concentration and moderate field condition.

To compare the effect of the chelating agents on film thickness, Figure 6 compares the normalized thickness of films deposited on both functionalized and non-functionalized substrates using a moderate field of 30 V/cm and a low stock solution concentration of 1 vol%. The normalized thickness was calculated by comparing the mean value of the film thickness on the functionalized substrates with respect to the film thickness on the non-functionalized substrates. Normalized film thicknesses were used to isolate the impact of the chelating agents and to control for batch-to-batch variability in EPD deposition from different starting suspensions. It can be seen that after comparing the three chelating agents, the normalized film thickness for the DPA experimental group (1.44 ± 0.2) is higher than that of the normalized film thickness of the NTA group (0.97 ± 0.1) (*p* = 0.024).

The increase in film thickness for the substrates functionalized with DPA and not NTA can be explained by the combined effect of the EPD conditions chosen and the relative strength of the coordination bonds that each chelating agent has with iron oxide nanoparticles. Though chelating agents containing carboxylic acid functional groups, such as NTA, have been used to chelate with iron oxide nanoparticles, their coordination bonds are labile and tend to be replaced with stronger interacting groups (Walter et al., 2015). As a chelating agent, DPA has shown improved coordination to iron oxide nanoparticles attributed to the orbital overlap from the six-membered ring of the catechol group (Boyer et al., 2010). Additionally, because the EPD solution is non-aqueous and at a pH of approximately 2, the DPA hydroxyl groups retain their hydrogens and do not polymerize as they typically would in more basic, aqueous conditions (Walter et al., 2015). Therefore, the strong interaction between the iron oxide nanoparticles and dopamine promotes thicker film formation.

**FIGURE 7:**
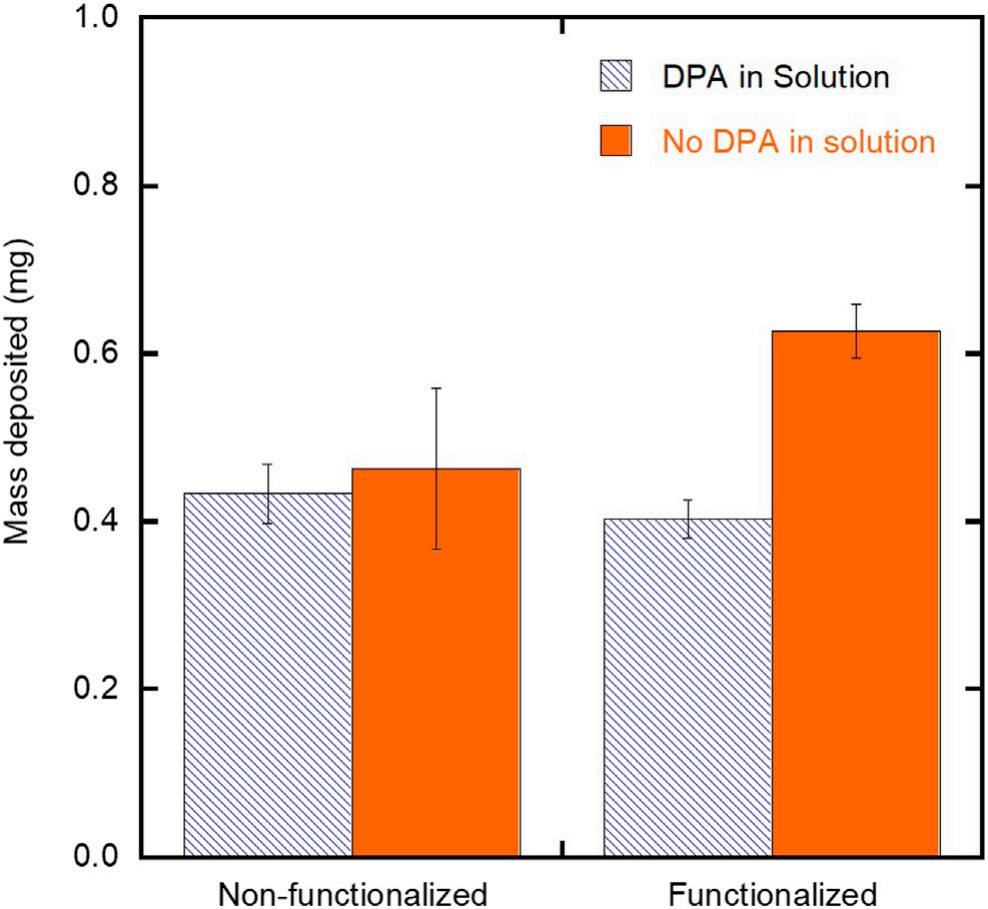
Effect of SH-PEG-DPA chelating agent in the suspension on the mass deposited for EPD on non-functionalized and SH-PEG-DPA functionalized substrates. The concentration of SH-PEG-DPA in the suspension was 0 or 0.5 g/L SH-PEG-DPA.

To confirm that the observed increase in film thickness (Figure 6) is the result of increased film substrate interactions and not due to the presence of any unbound chelating agents, two control experiments were performed. In these experiments SH-PEG-DPA was introduced into the suspension for deposition on both a functionalized and non-functionalized substrate under the low particle concentration and moderate field deposition conditions. SH-PEG-DPA was selected based on its ability to show an increase in film thickness under these deposition conditions onto a functionalized substrate (Figure 6). Figure 7 compares the mass of iron oxide nanoparticles deposited on both SH-PEG-DPA functionalized and non-functionalized substrates with SH-PEG-DPA in the suspension at either 0 or 0.5 g/L. For both non-functionalized and functionalized substrates, the presence of the chelating agent in suspension did not result in an increase in the mass deposited. The functionalized substrate with no chelating agent in the suspension yielded the highest mass deposited (Figure 7), confirming that the presence of chelating agent on the surface promotes increased deposition yield. The lack of increase in yield with SH-PEG-DPA in suspension is attributed to its relatively high molecular weight (3,400 g/mol), leading to steric hindrance between the deposited particles.

EPD deposition conditions also play a role in the ability to form thicker films. For example, the increased film thickness seen in the substrates functionalized with DPA can also be attributed to the chosen EPD parameters: low particle concentration and moderate field. At low stock suspension concentration (1 vol%), the solution is dilute enough to promote a well-dispersed suspension. Under these conditions, the particles that are moving towards the substrate for deposition are not agglomerated, resulting in the formation of a well-ordered and stable film (Besra and Liu, 2007). The moderate field (30 V/cm), in conjunction with the well-dispersed particles, avoids turbulence in the suspension that high fields produce and allows the particles to deposit with sufficient time to rearrange, forming a stable film (Amrollahi et al., 2015).

The other set of conditions for EPD that was used to observe the effect of substrate functionalization and EPD parameter choice on film deposition comprised of high stock solution concentration (5 vol%) and high applied field (80 V/cm). In this case, by using high particle concentration and increasing the applied electric field, the deposition of these films experienced particle agglomeration and more solution turbidity, along with less time for particle rearrangement after deposition (Amrollahi et al., 2015). After the particle films dried overnight, however, the films on the substrates that were not functionalized were highly unstable, with particles falling off the substrate at even the slightest agitation. Conversely, the films on the substrates that were functionalized with each of the chelating agents did not experience the same fragility, attributed to increased film-substrate interactions. However, due to the fragility of the films on the non-functionalized substrates, thickness measurements were not possible. These results qualitatively indicate that the use of substrates functionalized with chelating agents does improve the stability of the deposited particles. The observation of minimal loss of particles during handling of films deposited on functionalized substrates compared to the non-functionalized substrates indicates that there is a nominal increase in film-substrate adhesion through the use of chelating agents. The cross-sectional SEM images of films from functionalized substrates can be seen in Figure 5B.

**FIGURE 8:**
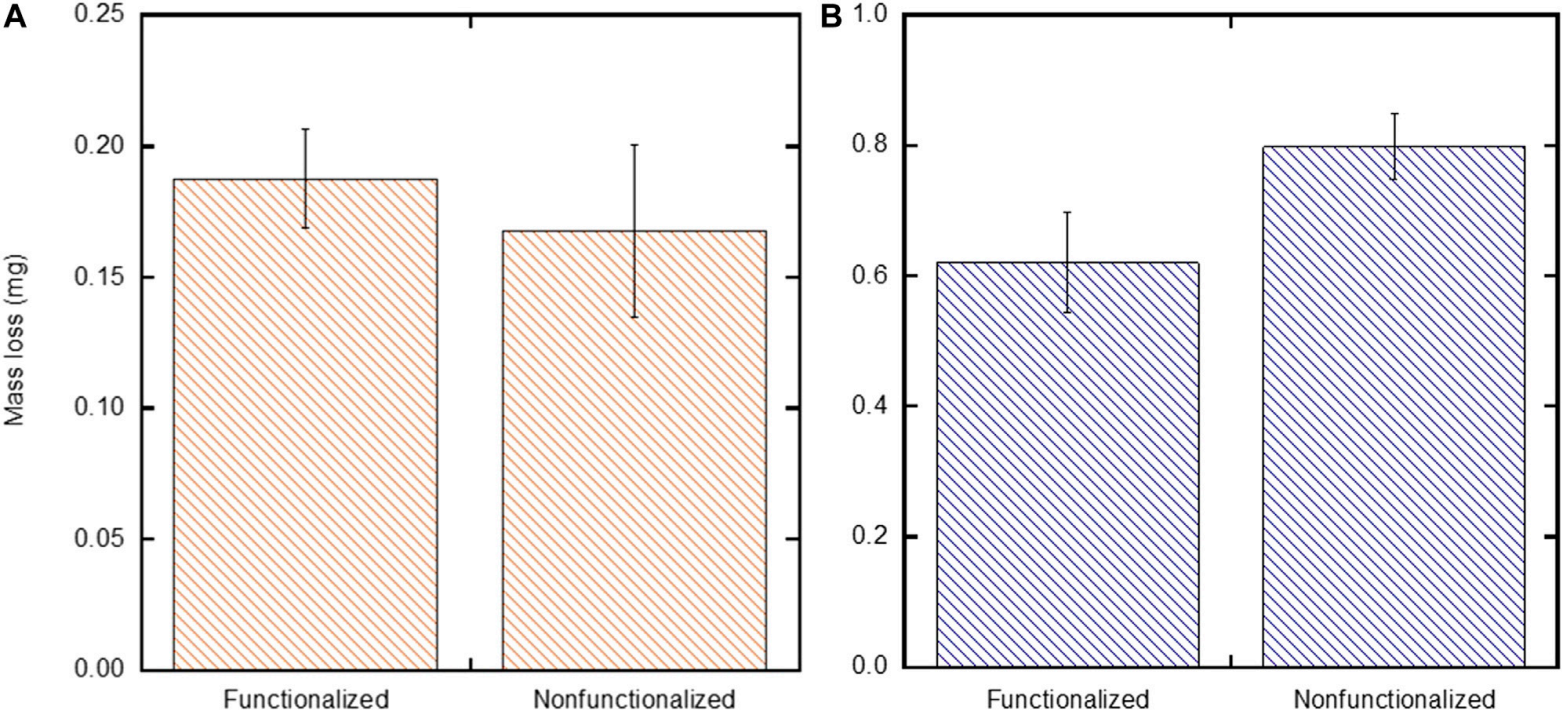
Comparison of mass loss between non-functionalized substrates and substrates functionalized with SH-PEG-DPA after the Tape Test (ASTM-D3359-17) was performed on films deposited with the **(A)** low particle concentration and moderate field and b) high particle concentration and high field conditions.

To assess the stability of the films on SH-PEG-DPA functionalized substrates when deposited under the two sets of EPD conditions (low stock solution concentration and moderate field, and high stock solution concentration and high applied field), the Tape Test (ASTM-D3359-17) was performed on these films, which is meant to assess the adhesion of films by applying and removing pressure-sensitive tape over cuts made into the film (ASTM, 2017). The mass of the deposited films after the adhesion test was compared to the as-deposited mass to determine mass loss (Figure 8). These results show that substrates functionalized with SH-PEG-DPA deposited with low particle concentration and moderate applied field lose a comparable mass (0.19 ± 0.02 mg) compared to those that are non-functionalized (0.17 ± 0.03 mg) (*p* = 0.34) (Figure 8A). However, Figure 8B shows that films on SH-PEG-DPA functionalized substrates that are deposited with the high particle concentration and high field condition lose less mass (0.62 ± 0.08 mg) than those that are non-functionalized (0.80 ± 0.05 mg) after undergoing the Tape Test (*p* = 0.01).

The lower average mass lost in films deposited with the low particle concentration and moderate field condition when compared to those deposited with a high particle concentration and field is expected, as a lower particle concentration and lower field is directly proportional to a lower yield (Eq. 1). However, when comparing the non-functionalized and functionalized substrates in the low particle concentration and moderate field condition, there was no significant difference in the mass lost between the two experimental groups. This is due, in part, to the conditions themselves traditionally leading to stable films due to a well-dispersed suspension (low particle concentration) and sufficient time for deposition and rearrangement at the substrate (moderate field). These results do suggest that further refinement is needed to improve the stability of functionalized substrates under these conditions. This refinement could include varying the molecular weight of the PEG spacer on the chelating agent molecule, which effects the arrangement of the molecules on the gold surface of the substrate (due to sterics) and therefore their ability to coordinate with incoming iron oxide nanoparticles.

The decrease in mass loss for SH-PEG-DPA functionalized substrates when compared to non-functionalized substrates under the high particle concentration and high field condition nominally supports the previously discussed qualitative observations of increased film stability in the cleaved, functionalized substrates (Figure 5B). However, with an appreciable amount of mass loss still present with the functionalized substrates under this set of conditions, an additional processing step to further increase adhesion may be necessary, such as flash sintering or another heat treatment (Dhiflaoui et al., 2017; Kwon et al., 2018). Varying the molecular weight of the PEG spacer on the chelating agent molecule, as mentioned above, could also be investigated to further improve film stability and decrease mass loss when tested with the Tape Test. Overall, these results show that chelating agents can be used to increase the stability of films deposited under less ideal EPD conditions.

**FIGURE 9:**
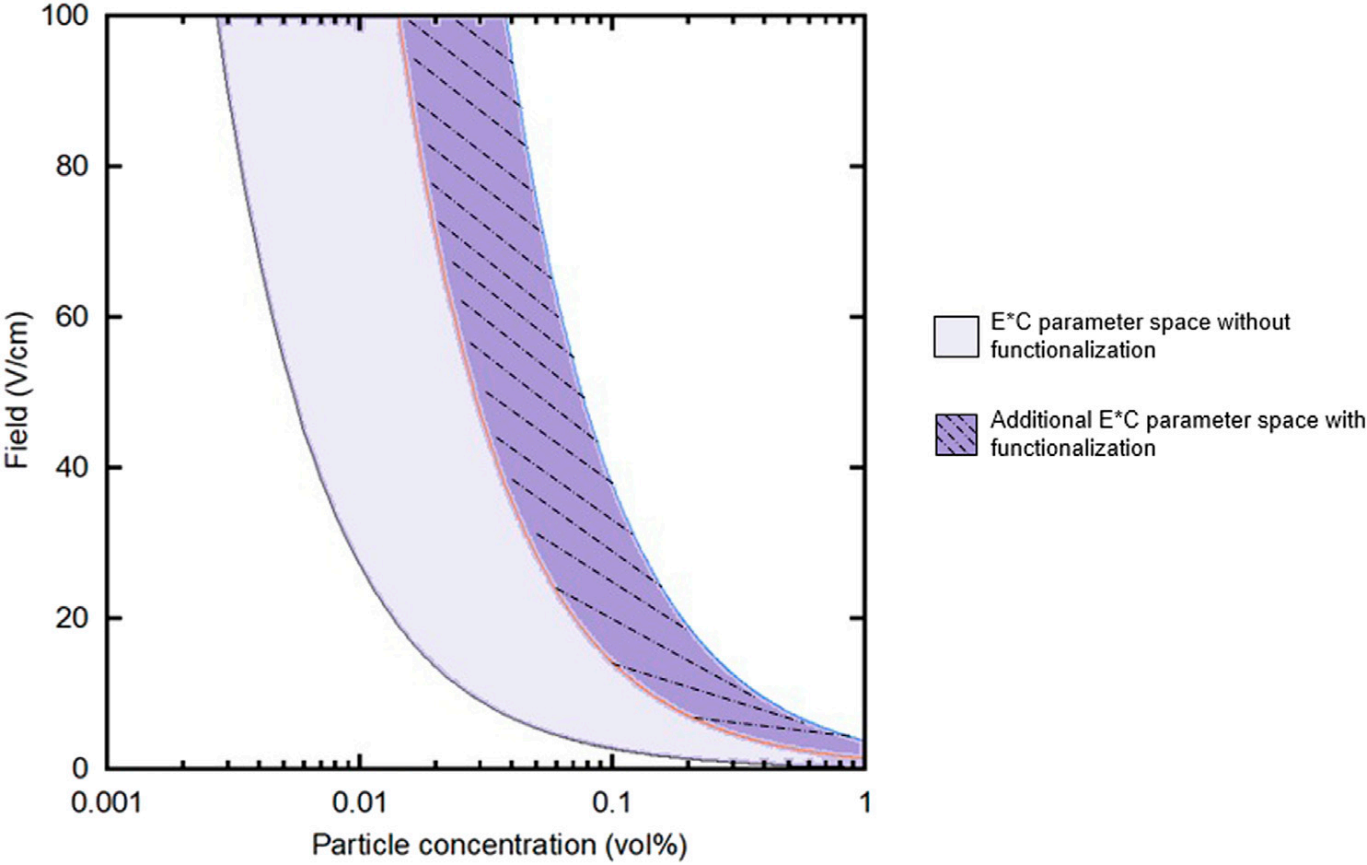
Field (*E*) vs. particle concentration (*C*) plot showing the E*C processing area, shaded in solid, for the parameter space used in this work. The solid-dashed line area represents the additional parameter space that is available by functionalizing substrates with chelating agents.

Functionalizing substrates with chelating agents for EPD has the ability to increase yield, stability, and thickness of films over a larger range of applied electric field and particle concentration than traditional EPD. In this study, two key deposition parameters were varied: applied external electric field and particle concentration. The achievable processing space could be bound by two limits: the constant field (*E*) and particle concentration (*C*) products (E*C) of the lowest and highest *E* and *C* values, shown in Figure 9. These values were determined based on the range of E*C products that yielded “good” deposits, the lower bound of which was determined based on our lab’s previous work (Mills et al., 2020). A “good” deposit was characterized as follows: consistent coverage over the entire substrate and minimal portions of the film flaking off after drying.

Without functionalizing substrates with chelating agents, this particular system’s processing space as represented in the field vs. concentration curve is smaller than if functionalized substrates are used. Given that there is a minimum applied electric field that must be applied to move the particles in solution and the minimum particle concentration was the same for both experimental groups, the non-functionalized and functionalized curves share the left-most curve. However, the functionalized substrates provide the opportunity to explore a larger processing space, specifically here higher particle concentrations and higher applied fields. This is represented by the increase in area between the two curves, the right-most curve shifting farther to the right. Functionalizing substrates for EPD with chelating agents, then, seem to overcome the practical challenges that increasing these two parameters, particle concentration and field, introduce to traditional EPD films.

## 4 Conclusion

A method to fabricate thick, stable films of nanoparticles has been developed for use in EPD by increasing film-substrate interactions. By functionalizing conductive gold substrates with chelating agents, these results preliminarily show that the deposited films of particles are thicker and have increased stability on the deposition substrate. The observed improvement in film deposition can be attributed to both the particular chelating agent and the conditions under which the EPD was performed. It was shown that the chelating agents that have stronger coordination and coordination bond stability (phosphonic acid and dopamine) to iron oxide nanoparticles increase film thickness and stability. Additionally, the conditions at which EPD is carried out with functionalized substrates also affects the film deposition by introducing more agglomerates (high particle concentration) or decreased rearrangement time (high field). Overall, surface functionalization of the substrate yields a larger parameter space to perform EPD with, providing the opportunity to fabricate thicker, more stable films. There are additionally opportunities to further enhance film stability and thickness *via* the investigation of the effect of altering the structure of the chelating agent molecule and additional processing steps. This is a versatile technique that could be used in different particle systems, providing a method to assemble thick films of metal oxide nanoparticles for a wide-range of applications. Ultimately, the films deposited on functionalized substrates could be implemented in devices to expand the method. Improved nanoparticle film performance *via* increased thickness and stability could be useful in a number of applications, such as sensing, wearable electronics, and energy storage.

## Data Availability

The original contributions presented in the study are included in the article/Supplementary Material, further inquiries can be directed to the corresponding author.
