# Supplementary material for: Chelating Agent Functionalized Substrates for the Formation of Thick Films via Electrophoretic Deposition
Source: Front Chem. 2021 Jun 17;9:703528. doi: 10.3389/fchem.2021.703528 (PMC8245681; doi:10.3389/fchem.2021.703528)
Supplement: Supplementary file 1 [file DataSheet1.docx]

**b)**

**a)**

**Figure S1.** Characterization of the iron oxide nanoparticles used in EPD. a) XRD pattern confirming the synthesis of inverse spinel iron oxide (reference iron oxide peaks indicated by boxes in (a). b) The zeta potential of the synthesized iron oxide nanoparticles as a function of pH.


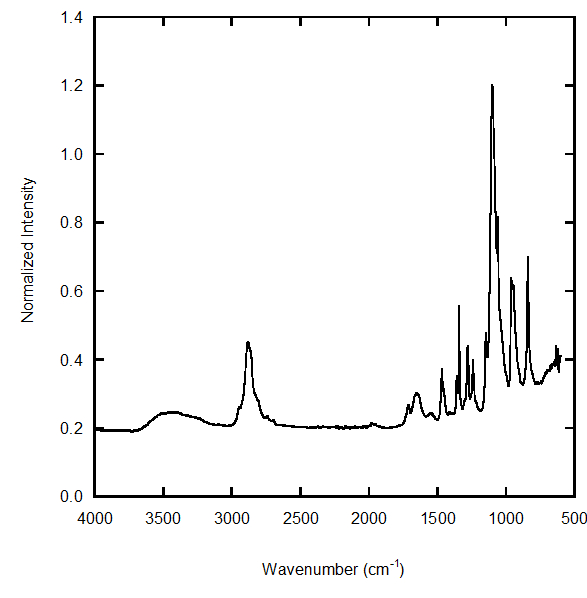


**Figure S2.** FTIR of the commercially available SH-PEG-NTA.

**b)**

Au 4f7

Au 4f5

**a)**

Au 4d5

Au 4d3

S 2p

C 1s

O1s

Au 4p3

Au 4p1

O KLL

**c)**

O 2s

**Figure S3.** XPS data for the gold substrate functionalized with a chelating agent. a) Survey XPS scan of a functionalized gold substrate. High-resolution multiplex scan of the S 2p peak (b) and C 1s peak (c).

DPA

PA


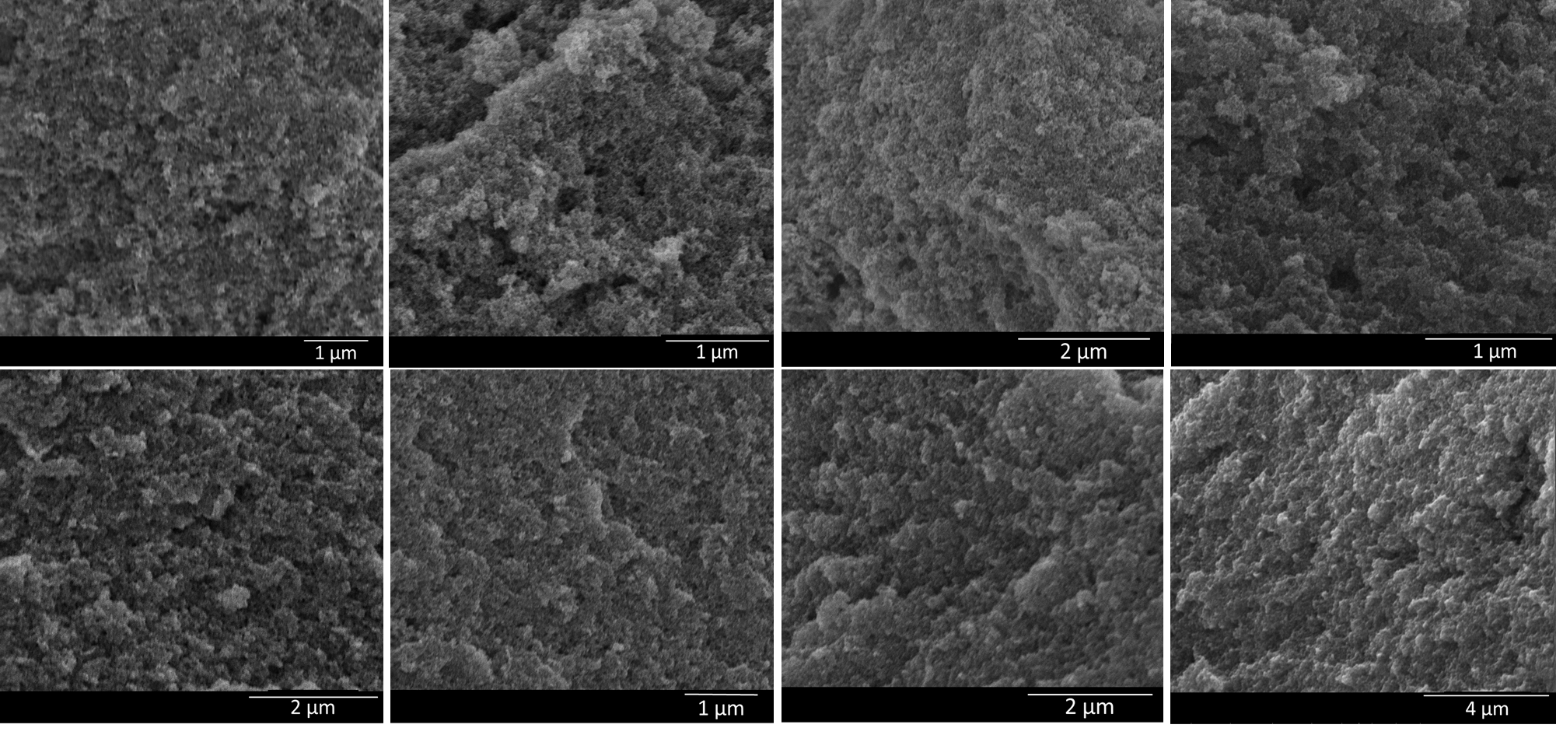


**Figure S4.** High-magnification SEM cross-sectional images of films comparing the non-functionalized films to each surface treatment group from the low particle concentration and moderate field condition.

NTA

Non-functionalized

Low particle concentration and moderate field
